# Supplementary figures and images for: The conserved transcriptional regulator CdnL is required for metabolic homeostasis and morphogenesis in Caulobacter
Source: PLoS Genet. 2020 Jan 21;16(1):e1008591. doi: 10.1371/journal.pgen.1008591 (PMC6994171; doi:10.1371/journal.pgen.1008591)

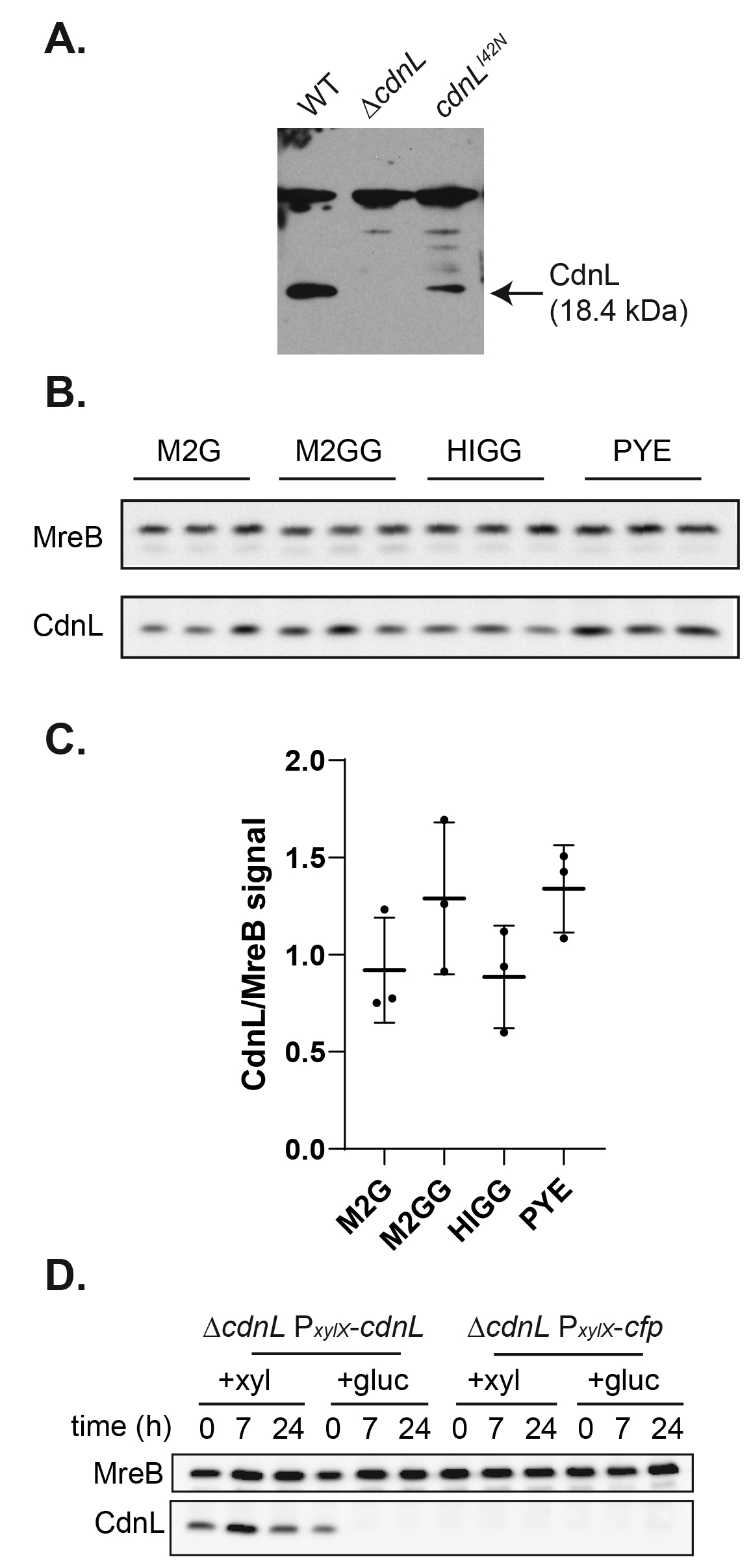

Supplement: S1 Fig — A. Immunoblot against whole cell lysates of the indicated strains (WT (EG865), ΔcdnL (EG1447), cdnLI42N (EG1416)) probed with CdnL antisera. B. Immunoblot against whole cell lysates of WT cells grown in indicated media. C. Quantification of CdnL levels from B. using ImageJ. CdnL values are normalized to MreB. Values for each condition are not significantly different from each other using one-way ANOVA with Tukey’s multiple comparison test. D. Immunoblot against whole cell lysates of ΔcdnL PxylX-cdnL (EG1403) or control ΔcdnL PxylX-cfp (EG3136) strains grown in PYE with xylose (xyl) or glucose (gluc) for the indicated times. CdnL is depleted within 7 hours of growth with glucose in EG1403. MreB was probed as a loading control. (JPG) [file pgen.1008591.s001.jpg]

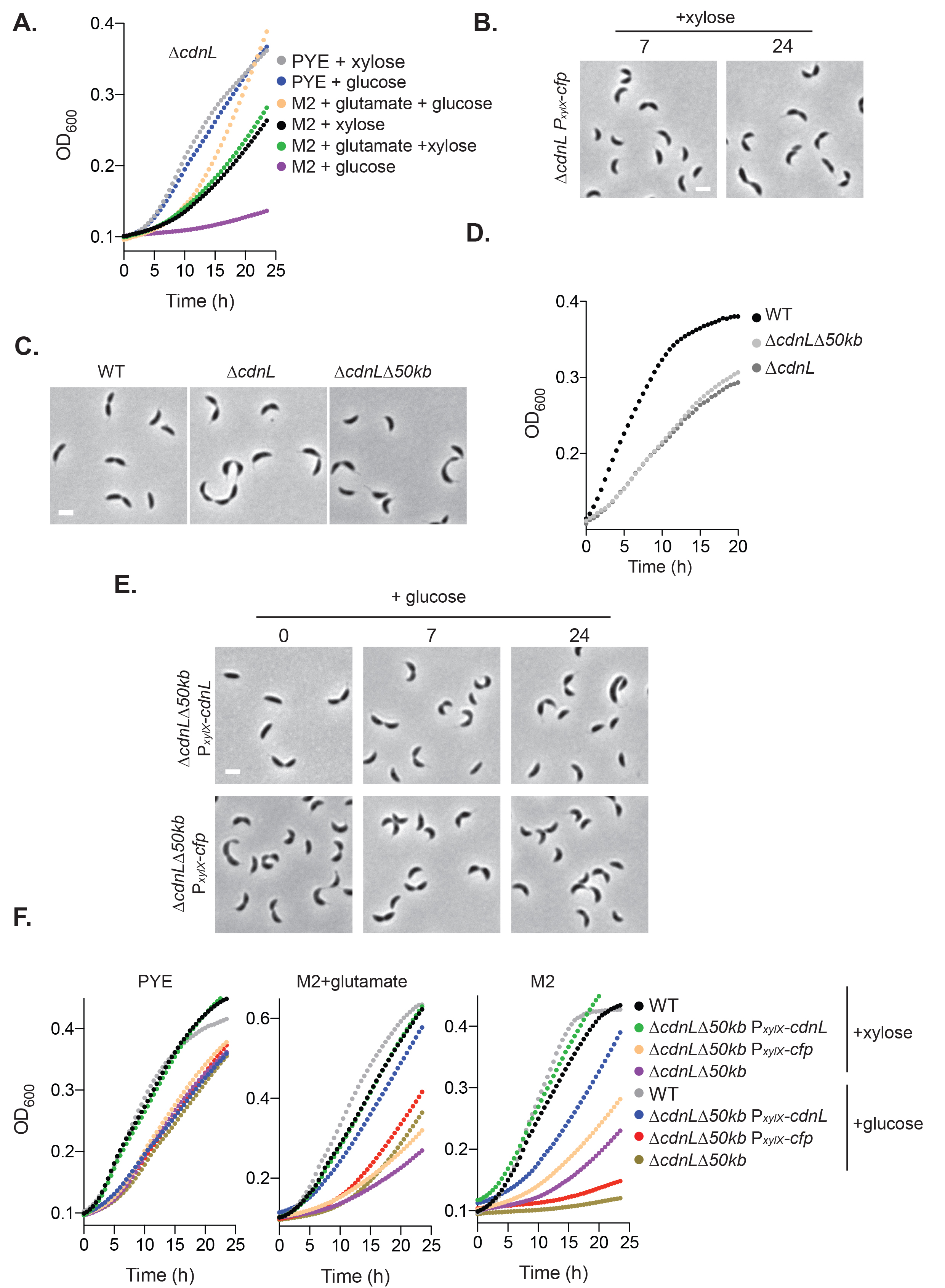

Supplement: S2 Fig — Growth curves of ΔcdnL cells (EG1447) in indicated media. B. Phase contrast images of ΔcdnLΔ50kb PxylX-cfp (EG3135) grown in PYE with xylose and imaged at the indicated time points. Time 0 is shown in Fig 2D. C. Phase contrast images and D. growth curves of WT (EG865), ΔcdnL (EG1447) and ΔcdnLΔ50kb (EG1415). Bar = 2 μm. E. Phase contrast images of ΔcdnLΔ50kb complemented with cdnL (EG3134) or cfp (EG3135) expressed from the PxylX promoter. Cells were grown in PYE xylose, washed, grown in PYE glucose, and imaged at indicated time points. F. Growth curve of ΔcdnLΔ50kb PxylX-cdnL and ΔcdnLΔ50kb PxylX-cfp grown in indicated media. Experiments were performed in triplicate and mean is shown. (JPG) [file pgen.1008591.s002.jpg]

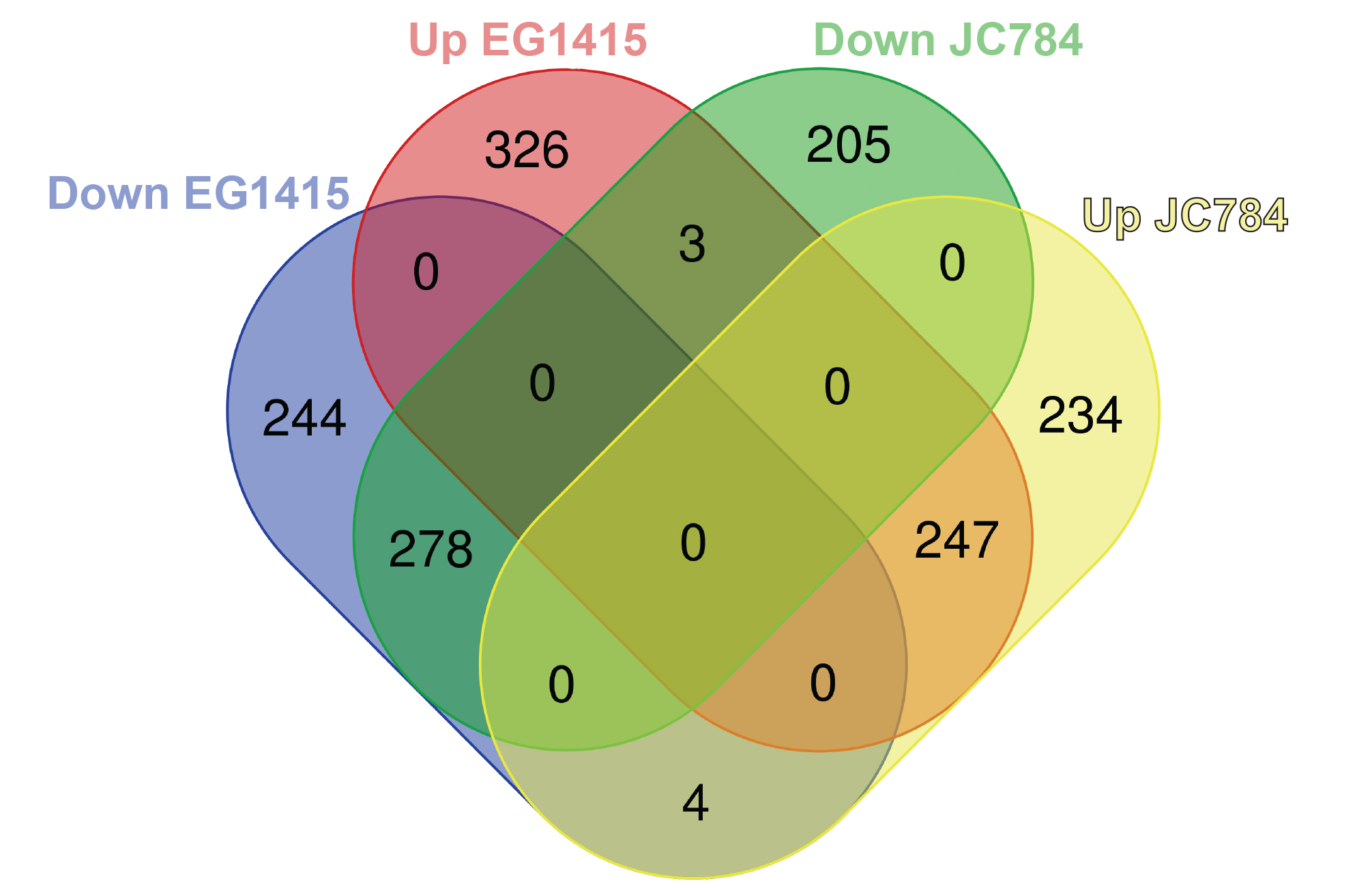

Supplement: S3 Fig — (JPG) [file pgen.1008591.s003.jpg]

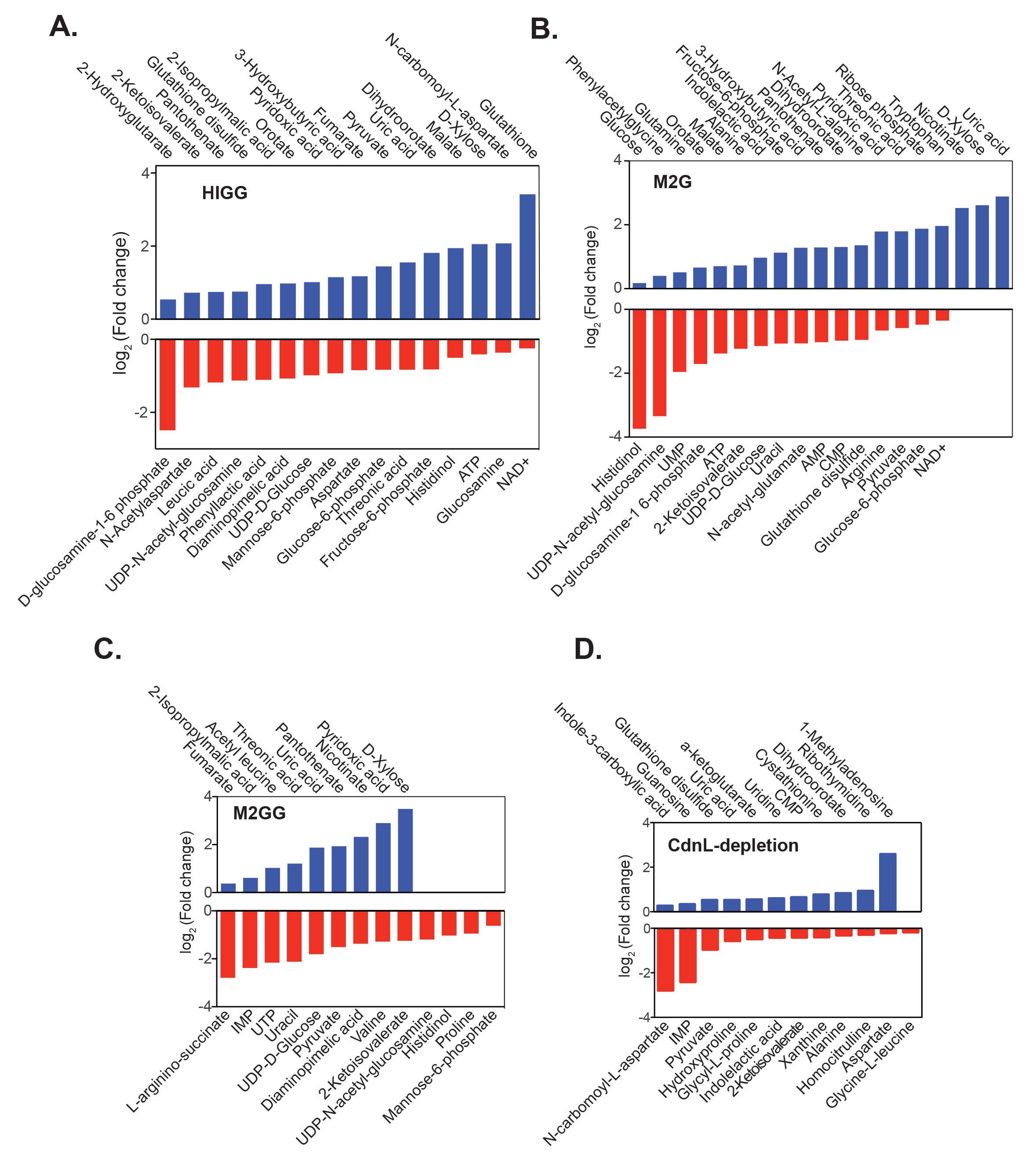

Supplement: S4 Fig — A-D. WT (EG865) and ΔcdnL (EG1447) cells were grown in indicated media until log-phase. B. Cells were grown in M2GG, washed and grown in M2G for 12 hours before extracting metabolites. D. EG1403 cells were grown without xylose for 8 hours to deplete CdnL before metabolite extraction. P < 0.05. (JPG) [file pgen.1008591.s004.jpg]

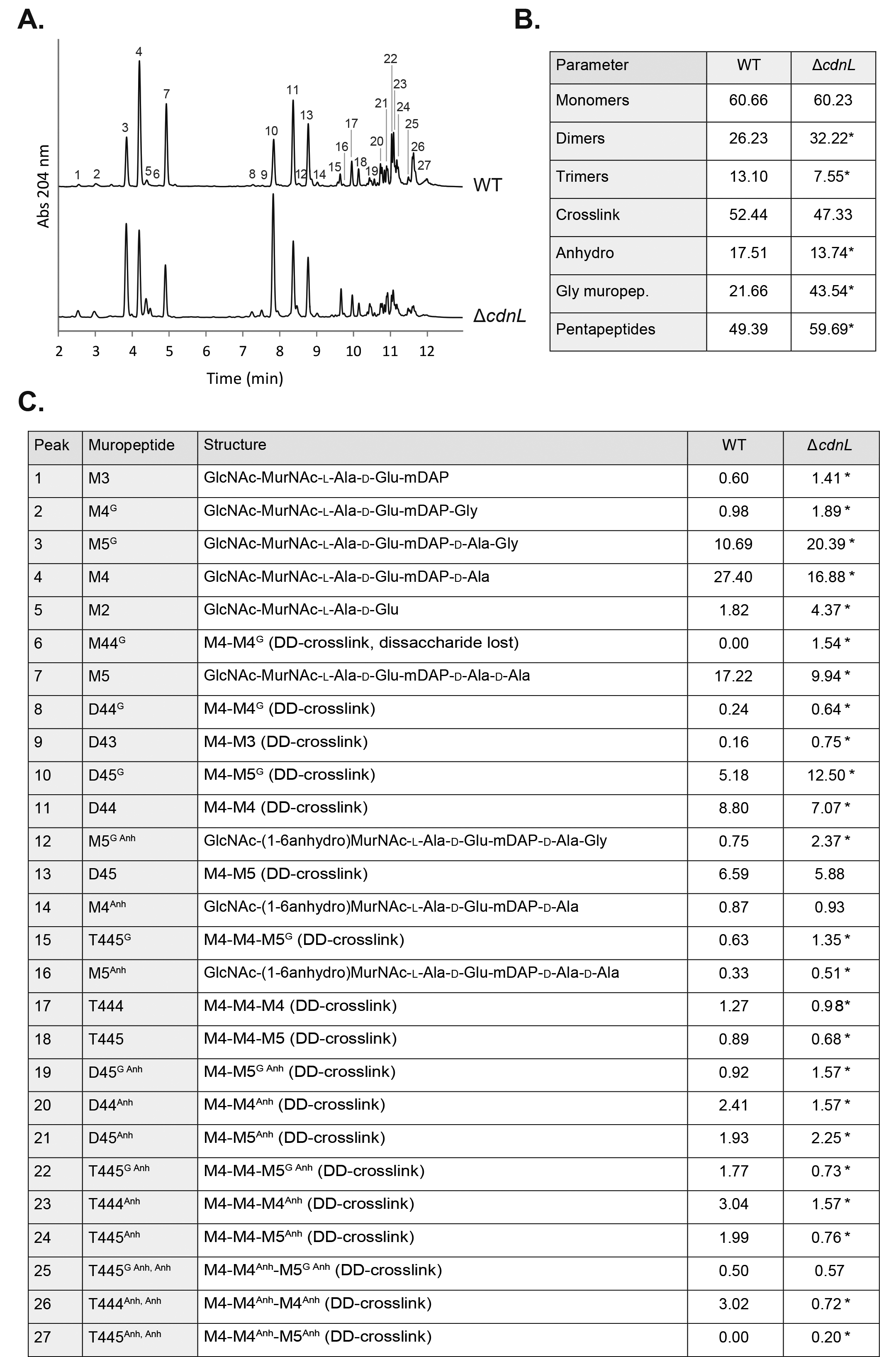

Supplement: S5 Fig — A. Representative chromatograms of the muramidase-digested sacculi of WT (EG865) and ΔcdnL (EG1447) cells. B. Relative molar abundance (%) of monomers, dimers, trimers, percentage of crosslinkage (proportion of crosslinked peptide side chains), muropeptides with a residue of (1–6 anhydro) N-acetyl muramic acid (Anhydro), Gly containing muropeptides (Gly muropep.), and pentapeptides. C. Muropeptide relative molar abundance (%). GlcNAc: N-Acetyl glucosamine. MurNAc: N-Acetyl muramic acid. Ala: Alanine. Glu: Glutamic acid. mDAP: meso-diaminopimelic acid. Gly: Glycine. Statistical analysis performed using t-test analysis. * = P < 0.05 and > 10% variation compared to WT. (JPG) [file pgen.1008591.s005.jpg]
