# Supplementary material for: The conserved transcriptional regulator CdnL is required for metabolic homeostasis and morphogenesis in Caulobacter
Source: PLoS Genet. 2020 Jan 21;16(1):e1008591. doi: 10.1371/journal.pgen.1008591 (PMC6994171; doi:10.1371/journal.pgen.1008591)
Supplement: Attachment — Submitted filename: Woldemeskel Response to Reviewers.pdf [file pgen.1008591.s013.pdf]

Responses to Reviewer Comments

Reviewer #1: This is a well-written and clearly presented manuscript describing the effect of deleting cdnL on Caulobacter cell morphology and metabolism. The morphology phenotypes of the cdnL mutants were very thoroughly characterized, and the interpretations tracing the phenotypes back to lipid II and PG limitation makes sense based on the data presented. In general, this manuscript is a thorough and important description of the global implications of loss of this critical transcription factor. There are a few points made by the authors where the data and discussion should be elaborated on to accurately and effectively support their conclusions, as detailed here.

We thank the reviewer for their careful reading of our manuscript and recognition of the importance of our findings.

1. The authors state that they became interested in CdnL through a screen for spontaneous suppressors of a dominant lethal mutant in FtsZ that has defects in PG metabolism. This is particularly interesting because of the phenotypes in the cdnL mutant strain related to PG. However, the authors never tie their findings with the cdnL mutant strain back to the original suppressor screen. Given what they know now, why do they think the point mutation in cdnL would suppress the FtsZ mutant? This is particularly confounding given that based on the data presented, one would expect that a mutation that destabilizes CdnL should result in defects in PG metabolism and it is not clear how this would suppress the defects in PG metabolism in the FtsZ mutant.

As the reviewer notes, we first identified CdnL as a regulator of morphogenesis in our screen for suppressors of FtsZΔCTL. We are pursuing the mechanism of ΔCTL suppression in a separate project in the laboratory but do not yet have a clear mechanism to propose. Indeed, as the reviewer notes, both deletion of *cdnL* and production of ΔCTL cause defects in cell wall integrity, which would lead us to predict they would exacerbate rather than suppress each other. The spontaneous ΔCTL suppressors we have identified are all in global regulators of transcription (*cdnL*, *phoB*, and *spoT*/ppGpp) leading us to think suppression requires modulation of numerous cellular activities. To address the reviewer's comment, we have added a paragraph in the discussion (lines 431-443) elaborating on the possibilities by which changes in metabolism that occur during *cdnL* loss may contribute to FtsZΔCTL suppression.

2. Line 206 – When the authors compare their RNA-seq vs. microarray data, they state that there is "significant overlap" between the two datasets. But based on the pie chart in supplementary figure 1, it looks like there are as many genes only differentially

expressed in one dataset as there are overlapping. Wouldn't that suggest that many of the results are specific to one dataset? Does that suggest that the missing 50kbp region has a large effect on the transcriptome? The authors also state they are using a Pearson correlation coefficient for significance, but that's just indicating that there is a linear relationship. Did the perform a hypergeometric test for over-enrichment between the two datasets?

Based on the reviewer's suggestion, we performed a hypergeometric test (specifically a Fisher's exact test of gene overlap) and found significant overlap between our transcriptomics data sets with a p-value of $2.2e^{-16}$. We have updated the results section to include this information (lines 207-210). Additionally, we have included a Venn diagram (Supporting Figure S3) that better visually represents the overlap between the two datasets in place of the pie chart we previously included. We still include the plot of fold change in transcript abundance in Δ*cdnL* by RNASeq vs microarray as it demonstrates that even in cases where the 2-fold threshold was not met, the direction of transcript abundance change is generally the same in both Δ*cdnL* clones and their corresponding transcriptomics analysis (now in Figure 3). Given that the two mutant strains were generated and cultured independently in different labs (Goley and Collier) and that the transcriptomics of each was performed with different technologies (RNA-Seq and microarray) and analyzed independently by different researchers, we would argue that the high degree of overlap indicates this is a true (and, with our "2-fold in both" cutoff, probably conservative) representation of the transcriptional changes associated with long term loss of CdnL. Finally, our complementation analysis of Δ*cdnL* and Δ*cdnL*Δ50kbp demonstrate that each is restored to WT upon expression of *cdnL* with respect to growth, morphology, and glutamate auxotrophy.

3. In Figure 1b, it looks like there are more cells in the cdnL panels undergoing or just finishing division. Is this something that can be quantified? Is there is a delay in the final stages of cell division?

Shape mode 1 from our PCA of cell shape reflects the length distribution across the population and therefore indirectly reports on cell division efficiency. In PYE, there are certainly some cells that become elongated or, more rarely, filamentous. In these cases, we see cells with extended division sites, perhaps reflecting a slowed rate of constriction (Figure 1b). However, the mean cell length is close to WT and in minimal media the length is actually shorter than WT with no obvious filamentation or defects in constriction or cell separation. Our PCA of cell shape also found 2 shape modes (modes 4 and 5) that reflect, in part, the degree of constriction (waist to pole width ratio, see figure below). In those shape modes, there is no difference in mean between WT and Δ*cdnL* cells grown in PYE. In mode 4, there are opposite trends for cells grown in

HIGG and M2GG (Δ*cdnL* appears less constricted in HIGG, more in M2GG). In mode 5, there is no difference in the mean value in any medium. Interestingly, in the CdnL depletion at 7 or 24 h without CdnL we do not see filamentation at all, and instead see cell shortening in PYE suggesting the potential division defects observed for Δ*cdnL* in PYE arise after long-term growth without CdnL (Figure 2). We also quantified the fraction of cells with midcell FtsZ or midcell fluorescent D-Ala labeling in PYE as an indicator of cell cycle status and actually found that fewer Δ*cdnL* cells had midcell Z-rings or midcell HADA incorporation compared to WT (see figure below). Since we did not observe a consistent defect in division across media conditions, and since in the CdnL depletion we do not see cell elongation, we opted not to include a discussion of these data in the manuscript.

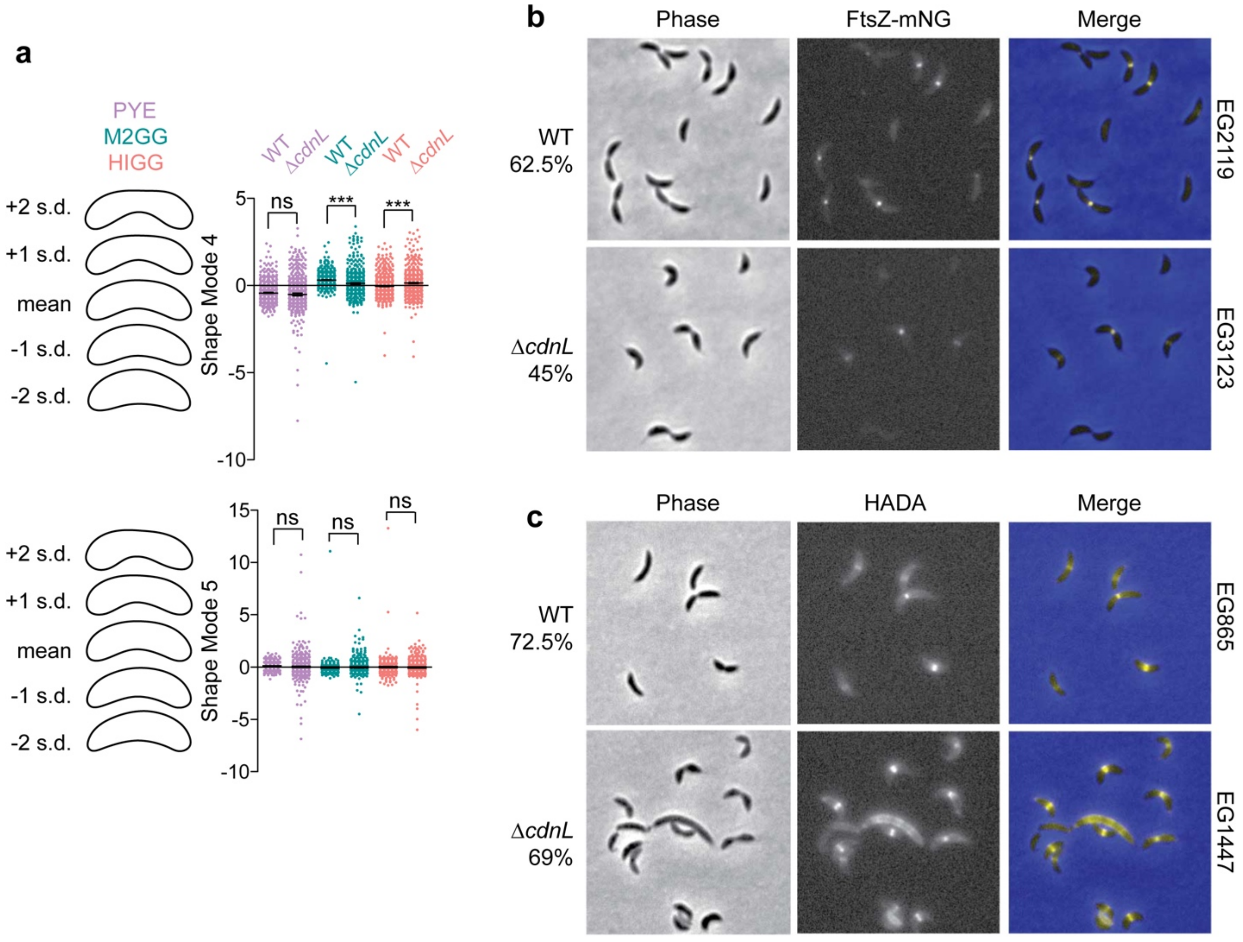

Figure above: **a**. Shape modes 4 and 5 from PCA of cell shape in WT vs Δ*cdnL* cells does not indicate a clear difference in constriction frequency in Δ*cdnL*. **b and c**. FtsZ localization and fluorescent D-alanine (HADA, 5 min pulse) labeling of active peptidoglycan metabolism in WT vs Δ*cdnL* cells grown in PYE. In each case, Δ*cdnL* cells show a reduced frequency of midcell localization (determined by manual counting and indicated below each strain name), suggesting fewer pre-divisional cells than WT.

4. It is difficult to interpret the MreB localization data in Figure 2. Does the more mid-cell focused dispersion of MreB along the length of the cell in the CdnL mutants mean that the MreB is mis-localized or does that mean that the cells are simply not elongating due to a lack of PG? Could an alternative explanation of the MreB localization be that more of the CdnL mutant cells are arrested in cell division or have defects in cell cycle progression, leading to persistent MreB localization to mid-cell? Is there a way to differentiate between these possibilities? The authors could analyze FtsZ localization and if the mutant cells are unable to complete fission or are stuck in a particular developmental stage, the proportion of cells with polymerized Z-rings should be different in the mutants. Some discussion of these other interpretations should be included.

Based on the comments we received from all reviewers, we have decided to remove the MreB, CtpS and CreS data from the manuscript since they were viewed as inconclusive and distracting from the main narrative.

The authors could also explain the assumptions that they make when selecting cells for FWHM fair. Can the WT and cdnL cells both be fit to the same Fourier series model? The authors could expand on how they use the demographs to select cells “based on cell length bins”?

Based on the reviewers comments we have decided to take out the MreB localization data.

5. Related to point 4, it is not clear how CdnL is affecting CtpS abundance or localization following induction of expression from a xylose inducible promoter.

Based on the reviewers comments we have decided to take out the CtpS data.

6. Figure 4B could be expanded to be more comprehensive. For example, the authors discuss the glyoxylate bypass in this section of the text, but it is not shown in the figure even though isocitrate lyase and malate synthase are two of the most up-regulated genes.

Thanks for the suggestion. We have added the enzymes and arrows (in green) indicating the glyoxylate pathway.

7. How are the authors normalizing the Lipid II and PG measurements in fig 5? If the measurements are normalized to per cell, could the differences in PG amount simply be due to the smaller size of cdnL cells?

For the lipid II and PG measurements, the same total OD units (roughly reflecting total cell mass) were used as starting material for different samples. Therefore, our measurements actually report on amount of lipid II or PG per OD unit. It is true that a change in the surface area (SA) to volume (V) ratio of the cell (caused by a change in cell size) could cause a change in total amount of surface-associated molecules like lipid II and PG. However, if Δ*cdnL* cells are smaller, we expect an increase in SA relative to volume and therefore a potential overestimate of surface-associated material in smaller cells.

Other comments:
1. The authors should show the data referenced as data not shown in Line 132.

We have removed MreB data discussed here from the manuscript.

2. The introduction and discussion should be updated to include the recently published expression profiling performed in Mycobacterium tuberculosis strains expressing CarD mutants.

Agreed. We have updated the manuscript and included the findings from the Mycobacterium CarD paper.

3. Lines 259-260, 264-265 – The authors should map those enzymes onto the figure

Done.

4. Figure 5-In fig 5a, the “WT” column seems unnecessary.

We have removed the WT column based on the reviewer’s comment.

5. Fig 5d is a little difficult to read because the black and grey of the WT vs. cdnL points are pretty similar looking.

We have updated the colors on the figure based on the reviewer’s comment.

6. Figure 4- Are the metabolites that are paired above/below in 4A linked in some way? If not, the figure should be presented differently, because it currently the figure implies a relationship between the metabolites.

Thanks for the suggestion. We have adjusted the figure so that upregulated and downregulated metabolites are separated.

Reviewer #2: This study by the Goley lab investigates the role of the transcriptional regulator CdnL in metabolism and morphogenesis in Caulobacter. The paper starts with a detailed phenotypic analysis of a cdnL deletion mutant with regard to cell shape, demonstrating that cdnL mutant cells are wider, more curved and have shorter stalks than the wild type. Furthermore, the authors demonstrate that the localization patterns of MreB and CtpS are mildly affected in the cdnL mutant. With the aim to explain the phenotypes of cdnL mutant cells mechanistically, the authors then monitor transcriptome and metabolome changes in the cdnL mutant (in comparison to WT) and search for mutations that are synthetic lethal with the cdnL deletion. These high-throughput analyses indicate that the cdnL mutation has pleiotropic effects and that in particular various metabolic pathways are affected by CdnL loss. Among other effects that are described in the paper, the authors show that cdnL mutant cells have lower levels of Lipid II, which according to the authors could contribute to the observed morphological defects.

Overall, this paper thoroughly describes the phenotypes and cellular consequence of loss of CdnL function. The authors make a number of interesting observations, however, most of these observations remain rather unconnected, leading to only limited mechanistic insight into CdnL functions on morphogenesis. The authors could have performed a number of additional genetic studies to better link their findings. The comments below include suggestions for such experiments as well as a number of other issues that need to be addressed.

We thank the reviewer for the careful reading and critique of our work.

Major comments:

1) Introduction: CdnL in Caulobacter has been studied in a previous study by Gallego-Garci et al. The authors should provide more background about the findings that were made in this study. Furthermore, more information about CdnL in other bacteria could be provided in the introduction.

We have updated our manuscript to better reflect the findings from Gallego-Garcia , 2017 and include additional information on CdnL homologs in other organisms (lines 48-67).

2) The authors compare the cdnL deletion mutant with wild type throughout the study. However, experiments showing that ectopically expressed cdnL can rescue the mutant phenotypes are not included. Given that the cdnL mutant strain E1415 harbors a 50kbp-deletion, such complementation assays are critical to confirm that the observed phenotypes are caused by absence of CdnL.

Thank you for bringing this important point to our attention. Based on the reviewers' comments we have included extensive complementation analysis for both Δ*cdnL* and Δ*cdnL*Δ50kbp strains (Figure 2 and Supporting Figure S2 and lines 137-181). We apologize for neglecting to address this critical point in the original version of our manuscript.

3) Related to point 2, it is possible that cells lacking CdnL compensate absence of the protein by indirectly up- or downregulating many genes. Indeed, the finding that 30% of the transcriptome is misregulated points to various indirect effects that CdnL loss has on gene expression. To analyse more directly the effects caused by loss of CdnL function the authors could deplete (or replete) CdnL and analyze changes in cell morphology, gene expression and metabolome as a function of time. Furthermore, ChIP-seq experiments would lead to valuable insight into the direct regulon of CdnL, which in turn is expected to lead to a more mechanistic understanding of CdnL function.

Based on the reviewer's comment, we have (1) analyzed the changes in cell morphology as CdnL is depleted over time (Figure 2d,e and lines 171-181) and (2) performed ChIP-seq with CdnL and RpoD antisera to identify CdnL's occupancy across the genome (Figure 3 and Table S3, lines 225-258). Moreover, we compared our transcriptomics dataset with the ChIP-seq and have now identified the direct regulon of CdnL. Indeed, as the reviewer predicted, most of the effects of CdnL loss on the transcriptome are indirect adaptations to the immediate impacts of CdnL loss.

4) Fig. 1a: WT Caulobacter cultures seem to grow equally fast in M2G and PYE medium during exponential growth, however, it is well established that the generation time is almost twice as long in M2G compared to PYE medium. The authors should comment on this.

It is true that when grown in test tubes or flasks with constant shaking, WT *Caulobacter* cells double in ~90 minutes in PYE and ~150 minutes in M2G. Our growth curves are performed in an automated fashion by growing cultures in 100 µL volume at 30 ˚C in a 96-well plate in a plate reader, with intermittent shaking only right before reading each time point. Under these conditions, we see that the doubling time of WT *Caulobacter* is much slower than we observe with constant shaking in a flask or test tube in PYE (~5 h)

and that the doubling time in M2G is, as the reviewer noted, similar to that of PYE under these conditions. We have added a note to this effect in the methods describing the growth curve experiments (lines 459-462).

5) Fig. 1a: The growth defects caused by the *cdnL* deletion are more severe in M2GG and HIGG medium than in PYE. Have the authors compared expression levels of cdnL under the different conditions?

To address the reviewer's comments, we grew WT cells in PYE, HIGG and M2GG and immunoblotted for CdnL. We found that CdnL levels are similar across all media conditions and have included these results in Supporting Figure S1 and lines 121-122.

6) Fig. 2: The outcome of the data describing the localization pattern of MreB should be better explained. The authors observe that MreB is hyperfocused in the mutant, but what do these data mean? Is hyperfocused MreB expected to lead to hypercurvature? Futhermore, the data in which the cdnL mutation is combined with different MreB point mutants (Fig. S2) are difficult to understand and the explanations provided by the authors quite vague (l. 166). The authors should try to provide more mechanistic explanations for these observations, or consider to remove these data to avoid confusion.

Based on concerns of all three reviewers with the relevance and clarity of our findings with MreB, we have removed this data.

7) Fig. 3: Similarly to the MreB data, the conclusions regarding CtpS localization data remain unclear. The authors observe that signal intensity of mCherry-CtpS filaments is reduced, but the protein level of CtpS is unchanged (as the authors state in l. 131 based on data not shown). In the discussion, they suggest that CtpS filament formation is impaired in the cdnL mutant as a consequence of lower CTP levels. However, have they ruled out the possibility that mCherry-CtpS levels are lower in cdnL cells compared to WT? Furthermore, it could be tested if modest CtpS overexpression or artificially increased CTP levels can alleviate the hypercurvature phenotype in the cdnL mutant.

In the course of revising this manuscript, our attempts to recapitulate the effects of *ctpS* overexpression reported in the literature failed to demonstrate the expected effect on cell curvature. Based on this confounding result and the reviewers' comments, we have removed the CtpS data.

8) The authors show that the levels of Lipid II are reduced in the *cdnL* strain and they suggest that this may contribute to the morphological and cell integrity defects of *cdnL*

mutants. However, experimental data testing the link between decreased lipid II levels and cell morphology are missing. Is it possible to directly assess the contribution of Lipid II misregulation to the morphological phenotypes, for example, by ectopically expressing the genes required for Lipid II synthesis in the cdnL mutant? In an alternative experiment, the authors could test whether artificial downregulation of Lipid II in an otherwise wild type strain phenocopies the morphology of cdnL cells. The authors should also discuss in more detail how reduction in Lipid II may affect the cell wall (l. 292). Is it expected to lead to more or less cross-linking, to longer or shorter polymers, etc? Similarly, the author could elaborate more on possible consequences of changes in the relative amounts of muropeptide species, e.g. Gly-containing muropeptides and pentapeptides (Fig. 5d).

Previously, other groups have shown that a decrease in lipid II levels causes an increase in cell width. In *Caulobacter,* Harris and Theriot (2016) lowered lipid II levels by treating with fosfomycin, an antibiotic that inhibits the initial steps of lipid II synthesis, and observed an increase in cell width. Furthermore, Irnov et al (2017) showed that *Caulobacter* cells without *hfq* have low levels of lipid II and shape defects including an increase in width. This defect was rescued by adding back the product of the cell wall enzyme that was downregulated in Δ*hfq.* We tried rescuing the decrease in lipid II levels in Δ*cdnL* by ectopically expressing *murB* and *murC* since these encode two of the first steps in lipid II synthesis. However, we were unsuccessful in increasing lipid II levels and rescuing shape defects. We suspect that this is because the substrates required for these enzymes, and many other metabolites, are also downregulated in Δ*cdnL* unfortunately precluding a simple test for sufficiency of lipid II to restore normal shape.

To address the reviewer's second point, we have added a paragraph elaborating on how loss of *cdnL* and the resulting decrease in lipid II levels may change PG metabolism and chemistry (lines 321-322 and 409-422).

9) The authors explain the observed glutamate auxotrophy of cdnL mutant cells with low amounts of alpha-ketoglutarate and downregulation of *gdhA* and *gltB*. However, it remains unclear whether misregulation of glutamate metabolism is linked to the morphological defects or if this is a separate phenotype of *cdnL* mutants. To address this question it would be interesting to analyze the morphology of cells cultivated in M2G, in which growth is arrested. For example, the authors could shift cells from M2G to M2GG, and then monitor changes in cell morphology over time.

Cells are unable to grow in M2G without glutamate. If we tried to cultivate them in M2G, they would not alter their morphology since growth is required for changes in shape.

However, since ∆*cdnL* cells are hypercurved, wider, and shorter than WT in both HIGG and M2GG media (which contain glutamate), glutamate is clearly not sufficient to prevent/restore those aspects of shape misregulation.

Minor comments:
10) L. 91: “an I42N mutation that disrupted CdnL protein stability”. In Fig. S1a the authors show that CdnL abundance is affected in this mutant, but not necessarily protein stability.

Thanks, we adjusted the text.

11) L. 123: A citation is missing supporting the statement that stalk elongation in response to phosphate starvation is distinct from developmentally regulated stalk biogenesis.

Thanks, we have added citations to support this claim.

12) L. 130-131: The experiments probing for changes in protein levels of MreB, CreS, FtsZ and CtpS levels should be shown as they are relevant for the interpretation of the findings shown in Fig. 2&3.

Based on the reviewers’ comments, we have removed the data relevant to cytoskeletal systems.

13) L. 178: “and found that cdnL mutant cells form fainter CtpS filaments compared to WT”. Do the authors want to say that the fluorescent signal of localized mCherry-CtpS was fainter than in WT. The wording of this sentence should be modified.

Based on the reviewers’ comments, we have removed the CtpS data.

14) L. 180: “mCherry-CtpS forms less robust filaments” What exactly do the authors mean with this statement? Is this really supported by the data?

Based on the reviewers’ comments, we have removed the CtpS data.

15) L. 236-237: “The presence of higher uridic acid across all media suggests that d_cdnL cells are preferentially catabolizing amino acids rather than carbohydrates as a carbon source”. The authors should double-check whether the suggested link between uridic acid and amino acid catabolism is correct. To my knowledge, uridic acid is the oxidation product of purine metabolism.

Uric acid is a byproduct of purine metabolism in many bacteria. Since purines are synthesized from glycine, glutamate, glutamine and aspartic acid, we extrapolated that a breakdown of purines will ultimately result in more amino acids being converted to purines. However, since there is not a direct link reported in bacteria between amino acid breakdown and uric acid in bacteria, we have removed this sentence from the results.

16) L. 274: “that genes required for lipid II biosynthesis” To help the reader, the gene names should be specified.

The genes are listed in Figure 5A with their corresponding transcript fold-change values. In the interest of readability, we opted not to list them in the text.

17) Fig. 1C: Please specify the unit of the y axis in the graphs shown in Fig. 1c.

Added.

18) Fig. 4a: Please check that the symbol “alpha” is correctly inserted, e.g. alpha-ketoglutarate, DL-alpha-Glycerophosphate.

Done.

Reviewer #3: The PG, shape and division defects have tremendous potential, and a variety of avenues are pursued (that sometimes) reinforce the PG defect model (like the MreB work) and at times takes us on an irrelevant and informative detour (like the CreS/CtpS work), but the most exciting discovery claiming that cdnL-I42N mutation suppresses the growth defect of the ftsZ-CC overexpression mutants is neither explored, nor validated (I found a cdnL-I42N plasmid in the strain list so I presume it was explored). There is no information about this mutant (how it was obtained), no backcrossing experiments, no description. On the basis of this result the authors embark on a re-evaluation the cdnL deletion phenotype, previously reported to have strong division and shape defects (doi: 10.1038/srep43240).

As the reviewer notes, we first identified CdnL as a regulator of morphogenesis in our screen for suppressors of FtsZΔCTL. We are pursuing the mechanism of ΔCTL suppression in a separate project in the laboratory (with the suppressor screen to be described in full in a corresponding manuscript) but do not yet have a clear mechanism to propose. To address the reviewers’ comments, we have added a paragraph in the

discussion elaborating on the possibilities by which changes in metabolism that occur during *cdnL* loss may contribute to FtsZΔCTL suppression (lines 431-443).

In addition, we would like to point out that in the prior, excellent report on CdnL function in *Caulobacter* (Gallego-Garcia et al 2017), the focus was on investigating CdnL essentiality, protein levels, interaction with RNAP, and regulation of rRNA transcription. In that paper, the authors showed a few cropped images of Δ*cdnL* or CdnL-depleted cells and briefly noted that loss of CdnL causes "cell filamentation" and "cell division defects" without any quantitation of morphology, as this was not the focus of their study. Our comprehensive shape analysis under multiple growth conditions in both deletion and time-course depletion experiments significantly add to our understanding of the impact of CdnL loss on cell shape in *Caulobacter.* Indeed, we observe mild cell elongation that is limited to Δ*cdnL* grown in PYE and that the most prominent and conserved effects of CdnL on cell shape are increased cell curvature, decreased cell length, and increased cell width.

Importantly, no complementation of the cdnL deletion phenotype is done, and this mutant (EG1415) turns out to have a large deletion elsewhere as was hinted by RNA-seq experiments and then later confirmed. Has any complementation be done to show that phenotypes are caused by the absence of CdnL? All important phenotypes must be tested for their ability to be corrected by providing CdnL in trans in the cdnL deletion mutant background.

We apologize for neglecting to include complementation experiments in our original manuscript, which we agree is critical for interpretation of our data. We have included extensive complementation analysis for both Δ*cdnL* and Δ*cdnL*Δ50kbp strains (Figure 2 and Supporting Figure S2, lines 137-181). In short, all of the phenotypes tested are restored to WT when *cdnL* is expressed in the Δ*cdnL* background and the 50kbp deletion does not impact any of the phenotypes we tested with or without *cdnL* expression.

Were experiments in figure 4 done with EG1415? It should be noted in the figure legends. What about the cell wall analysis in Fig 5 and Fig 6 that were done in EG1415? What are the effects of the 50 kb deletion and has the analysis been done with a complemented mutant? Which mutant was used in Fig S4-S6? The authors have made ΔcdnL, xylX::cdnL (EG1403) strain but there is no mention of it in the text and only used in supplementary Table S3 but the table seems incomplete and the date is not interpreted just dumped as Supplementary table can the authors show a (supplementary) figure illustrating if the complementation actually work and compare it between various cdnL mutant and complemented strains.

As stated in the original version of our manuscript, EG1415 was only used in the RNA-Seq experiment. After we determined from the RNA-Seq that EG1415 carried the 50kbp deletion we performed all other experiments with EG1447. The strains used for each experiment are listed in the figure legends and/or in the figures.

The glutamate auxotrophy of the cdnL mutant is novel and exciting, and there seems to be an obvious link with reduced pyruvate levels reported by metabolomics. If so, pyruvate and alpha-ketoglutarate should improve growth of cdnL cells. Is this the case??

We agree there is an obvious link from the TCA cycle through alpha-ketoglutarate to glutamate synthesis. However, we aren't certain what this experiment would show, since *gltB* (required for synthesis of glutamate from alpha-ketoglutarate and glutamine) is 4-fold downregulated in Δ*cdnL* (noted in the results section describing the metabolomics analysis). A mechanistic understanding of the apparently large scale changes to metabolic pathways and their kinetics in Δ*cdnL* would require an in depth flux analysis beyond the scope of this work.

Overall, there are some interesting links reported here which I think should be published in PLOS genetics but the major shortcoming is that the work is still very phenomenological and it is not clear how the molecular function of CdnL as general RNAP binding protein can lead to such specific PG and auxotrophic defects. Why are certain genes affected by the absence CdnL? Direct vs indirect effects should be dissected or at least some attempts should be made.

At the suggestion of the reviewers, we have now included ChIP-Seq of CdnL and RpoD to assess the direct vs indirect effects of deleting *cdnL* on the transcriptome. These data are now included in Figure 3 and Table S3 and described in lines 225-258.

For now, this has not been done, and as far as I could tell without complementation analysis described for most phenotypes, it's not even clear to me what is really caused by inactivation of cdnL versus possible unanticipated (unrelated) genetic defects in the mutant.

These concerns have hopefully now been addressed through our complementation analysis in Figures 2 and S2.

The MreB and CtpS work just merely reinforces the notion that something about PG metabolism is going wrong in the cdnL mutant, but why the transcript of PG metabolism is reduced and not MreB and CtpS itself or other genes really remains a mystery.

At the suggestion of the reviewers, the MreB and CtpS data have been removed.

Moreover, the authors don't comment on the FtsZ connection (even in the discussion), despite that there are very obvious models that can be proposed/discussed in the context of their cdnL mutant characterization which would make the story interesting and add a new twist that has not yet been reported.

We have added a discussion of the suppression of FtsZΔCTL toxicity by Δ*cdnL* to the manuscript. However, as mentioned above, we are pursuing mechanisms of suppression of ΔCTL by Δ*cdnL* in separate project in the lab, and do not yet have a clear answer. If the reviewer has specific suggestions for the models we should test, we would welcome them and be very happy to test them.

Since the shape and division defects of cdnL were previously already known, this manuscript contributes the metabolism (glutamate auxotrophy) and lipidII defects of the cdnL mutant, if indeed these phenotypes disappear when CdnL returns. This is a solid basis, but the story should be developed.

Again we would argue that our comprehensive and quantitative analysis of the shape defects of Δ*cdnL* go well beyond the description of “cell division defects” associated with Δ*cdnL* previously. In response to the reviewers’ concerns, we have now demonstrated through complementation analysis that the shape and growth rate defects and glutamate auxotrophy in Δ*cdnL* are attributable to loss of CdnL specifically.

Additional comments:

The claim that mreB localization is important to cdnL cells has not been shown. The mreB alleles may be partial loss of function (isolated as A22 MPP265 resistant mutants) that also affect localization. There is no causality to be inferred here, localization and activity cannot be separated here. The bottom line is that any stress on the PG pathway will be problematic to cdnL cells, including capsule or other mutations as the authors showed. The MreB and CreS/CtpS parts are not that helpful in understanding the problem with PG precursor biosynthesis.

We have removed the MreB, CtpS, and crescentin data and focused on identifying the CdnL direct regulon and complementation assays to ensure that the phenotypes observed when *cdnL* is deleted occur because of loss of CdnL.
